# An in-depth discussion of cholesteatoma, middle ear Inflammation, and langerhans cell histiocytosis of the temporal bone, based on diagnostic results

**DOI:** 10.3389/fped.2022.809523

**Published:** 2022-08-09

**Authors:** Bo Duan, Li-Li Pan, Wen-Xia Chen, Zhong-Wei Qiao, Zheng-Min Xu

**Affiliations:** ^1^Department of Otolaryngology-Head and Neck Surgery, Children’s Hospital of Fudan University, Shanghai, China; ^2^Department of Radiology, Children’s Hospital of Fudan University, Shanghai, China

**Keywords:** PyTorch, cholesteatoma, langerhans cell histiocytosis, computed tomography (CT) scan, deep learning

## Abstract

**Objective:**

This study aimed to conduct an in-depth investigation of the learning framework used for deriving diagnostic results of temporal bone diseases, including cholesteatoma and Langerhans cell histiocytosis (LCH). In addition, middle ear inflammation (MEI) was diagnosed by CT scanning of the temporal bone in pediatric patients.

**Design:**

A total of 119 patients were included in this retrospective study; among them, 40 patients had MEI, 38 patients had histology-proven cholesteatoma, and 41 patients had histology-proven LCH of the temporal bone. Each of the 119 patients was matched with one-third of the disease labels. The study included otologists and radiologists, and the reference criteria were histopathology results (70% of cases for training and 30% of cases for validation). A multilayer perceptron artificial neural network (VGG16_BN) was employed and classified, based on radiometrics. This framework structure was compared and analyzed by clinical experts according to CT images and performance.

**Results:**

The deep learning framework results vs. a physician’s diagnosis, respectively, in multiclassification tasks, were as follows. Receiver operating characteristic (ROC) (cholesteatoma): (0.98 vs. 0.91), LCH (0.99 vs. 0.98), and MEI (0.99 vs. 0.85). Accuracy (cholesteatoma): (0.99 vs. 0.89), LCH (0.99 vs. 0.97), and MEI (0.99 vs. 0.89). Sensitivity (cholesteatoma): (0.96 vs. 0.97), LCH (0.99 vs. 0.98), and MEI (1 vs. 0.69). Specificity (cholesteatoma): (1 vs. 0.89), LCH (0.99 vs. 0.97), and MEI (0.99 vs. 0.89).

**Conclusion:**

This article presents a research and learning framework for the diagnosis of cholesteatoma, MEI, and temporal bone LCH in children, based on CT scans. The research framework performed better than the clinical experts.

## Introduction

Middle ear inflammation (MEI) is a common condition among children. The incidence of otitis media is 8.1% (1), and imaging is not typically required in cases of MEI. However, for patients with MEI who present with hearing loss, and where the tympanic membrane cannot be visualized, imaging may be required (2). The specific imaging features will vary depending on the form.

The incidence of congenital cholesteatoma of the middle ear, once thought to be relatively rare, is on the rise and presently accounts for 2–5% of all the cholesteatomas (3). Langerhans cell histiocytosis (LCH) is a relatively rare condition that primarily affects children.

Soft tissue sarcoma and rhabdomyosarcoma are the most common among infants and young children. The head and neck are relatively common parts of the body involved in this condition; the percentage of cases with temporal bone involvement is approximately 7%.

Currently, high-resolution CT (HRCT) is typically used for the diagnosis and preoperative examination of suspected temporal bone tumors in clinical patients (4). Chronic otitis media and temporal bone tumors are more prone to erosion around the bone structure, which may require surgical treatment. Cholesteatoma is generally manifested in the location of the tympanic sinus (5). These two entities may appear in different positions of the tympanic chamber, which will also limit HRCT and differentiation between them.

Techniques that may be helpful for diagnosing tumors in the temporal bone include diffusion-weighted imaging or contrast-enhanced MRI; however, these methods may also affect the diagnostic results due to the high level of protein fluid present in inflammatory tissue and cases of otitis media (6).

Early stage/small tumors may not always be easily identifiable. In the medical field, a final accurate diagnosis must currently include an analysis of surgical tissue histopathology. Accurate preoperative differentiation between the two above-noted diseases is of significant clinical importance because most cases of temporal bone tumors require surgical treatment; chronic otitis media, on the other hand, can typically be treated conservatively (7). In addition, one of the key factors that can influence the choice of surgical methods and techniques is the available preoperative information concerning underlying diseases. Enhancing the imaging modality for identifying temporal bone tumors and otitis media can, thus, have a significant impact on clinical medicine, and efforts should be made to achieve this.

Radiomics is a method that is applied in the field of medicine that uses data representation algorithms to extract a large number of features from medical images. These features are known as “radiographic” data and can be used to observe several disease characteristics that are invisible to the naked eye. A radiology approach considers that the signal intensity patterns in medical images are closely related to the genetic, molecular, and biological characteristics of the tissue. These methods include T-scans and MRI scans.

The focus of the current study was to develop a deep learning framework for the automatic CT scan diagnosis of temporal bone diseases in children. To date, the radiographic features that are used to assess the utility of temporal bone disease have been rarely studied in the context of imaging. In this context, the current study aimed to provide a better analysis of these image scans to provide a more accurate diagnosis. The performance of artificial intelligence (AI) network model was tested on a separate dataset. The study also compared clinical specialists, including otologists, otolaryngologists, and radiologists.

## Methods and materials

### Study participants

The Institutional Review Committee of Children’s Hospital of Fudan University approved the current study. In procedural terms, a search of the clinical database identified 161 children who had undergone middle ear surgery between May 2013 and October 2020. Medical records were rigorously checked to exclude patients who had received CT scans and were subsequently diagnosed with acute otitis media or inner ear disease, and who lacked temporal bone. A final group of 119 patients was included in the collected retrospective data.

### Computed tomography imaging

**FIGURE 1:**
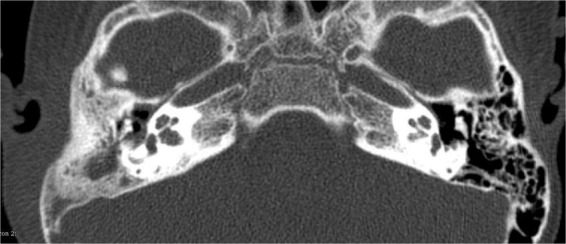
LCH of the temporal bone. The bone has not been destroyed.

All the registered patients required one or more CT scans of the temporal bone, and a total of 119 scans were available for analysis. These temporal bone scans were obtained using a 128-channel multidetector Somatom Definition Edge with z-Sharp™ Technology CT scanners. The procedure for conducting each scan was as follows. A full scan was required for each patient from the lower edge of the external auditory canal to the upper edge of the petrous bone. The collimation was set at 128 mm × 0.625 mm, the field of view was 220 mm × 220 mm, the spacing was 0.8 mm, the matrix size was 512 × 512, the voltage was 120 kV, 240 mass, and the axial section was 0.625 mm. The scans were performed on sections where lesions were present, and the number of axial CT sections per scan ranged from 30 to 50. The total number of scans performed was 2,588. All the images were downloaded from the doctor’s workstation and saved in the prescribed 224 × 224-pixel jpg format for subsequent analysis (Figure 1).

### Clinical labeling

The distribution of clinical labels for each ear was first arranged according to each patient’s diagnostic record. All the participants’ medical records were examined independently by two experienced otolaryngologists and radiologists, who, concurrently, resolved any differences that arose to reach a consensus. The facts that were selected in the training and testing sessions for the classification network were reflected in these clinical labels. On closer examination of these labels, the three most common types of cases were found to be “cholesteatoma,” “MEI,” and “LCH.” These three categories were based on web-based training and required in-depth learning to achieve adequate performance. The present article will summarize the distribution of clinical labels at a later stage. The labels for ear surgery were allocated according to pathology.

In temporal bone CT images, the lesion area accounted for a relatively small proportion of the overall image. During the training of a deep learning network, as the number of layers increased, additional lesion information was at risk of becoming lost in the image (8). Consequently, the current authors adopted a neural network with fewer layers.

**FIGURE 2:**
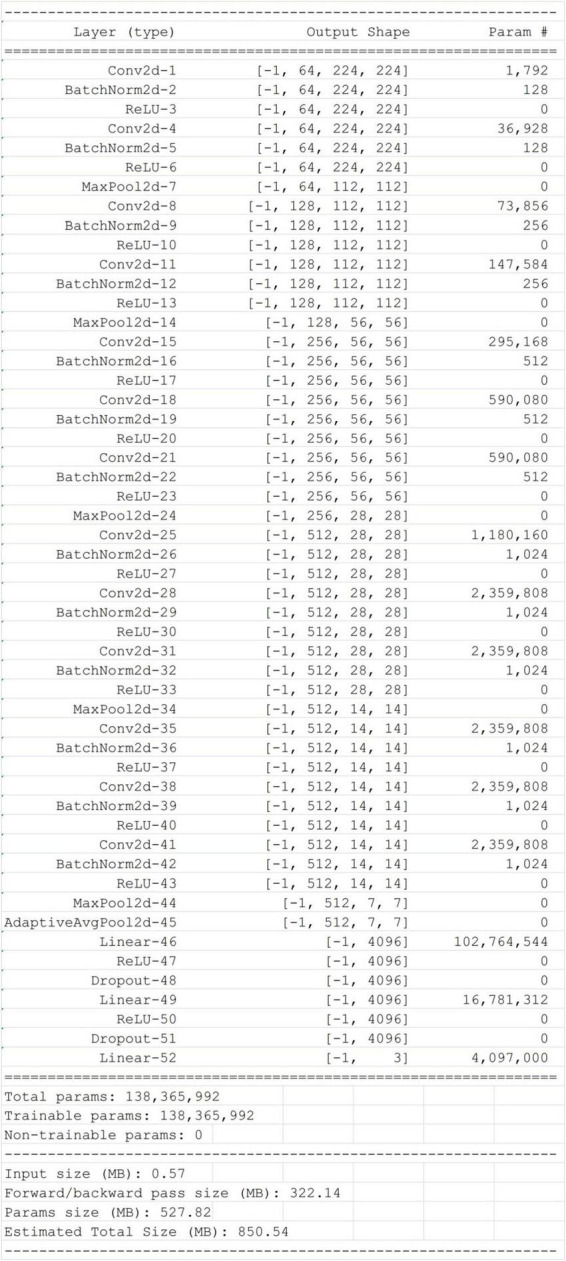
VGG16_BN structure diagram.

A convolutional neural network known as VGG16 (9) is an architecture with 16 layers of weights and 3 × 3 filters. After the convolutional layer, there are two interconnected layers, followed by the soft maximum output. The VGG16_BN network adds a batch normalization function to the VGG16 network. The matrix is then added to the network and outputted as three categories (see Figure 2). It has about 138 million network parameters.

### Data enhancement technology of the classification task

To ensure the efficiency of deep learning, all the images required a mirror image, minor rotation, cropping, scaling, and side-length moving to increase the training data for the classification task. The implementation model of the data volume enhancement was carried out in real-time by the classification network. Finally, the Bicubic interpolation algorithm was used to scale all the images to 512 × 512-pixel size.

### Classification task of the classification network

Based on the basic VGG16_BN classification network model, the Softmax function acted as its final classification layer. The probability distribution of cholesteatoma otitis media was trained using the PyTorch application in Python. A random selection of 85% of the dataset (*n* = 2,070) was used during the validation process, and five different parameters were included. The duration value of the training course was set to 100 (from the initial 0.01-bit speed). The remaining 15% of the data (*n* = 388) were stored and could be used to evaluate the performance of the model after the training was complete. Clinical experts could also use these datasets to assess performance.

### Professional performance evaluation

Four clinical specialists were selected to evaluate the diagnostic performance of their test datasets. Two of the specialists were registered otologists with 15 years of combined experience, and the remaining two specialists were radiologists trained in ear diseases with 16 years of combined experience. Each of the four experts could only select one of the three categories, based on the CT images, to diagnose a case.

### Analyzing and compiling statistics

To accurately evaluate the diagnostic ability of the AI model, the performances of the deep learning algorithm and clinical experts during data testing were summarized using the confusion matrix method, i.e., binarizing all the labels of the AI model and drawing the receiver operating characteristic (ROC) curve. The prediction probability was applied to measure the area under the ROC curve of the deep learning algorithm, and a 95% CI was estimated using the Tak Long Estate Algorithm. Python and Excel were used to conduct all the statistical analyses (alpha level, 0.05).

## Results

**FIGURE 3:**
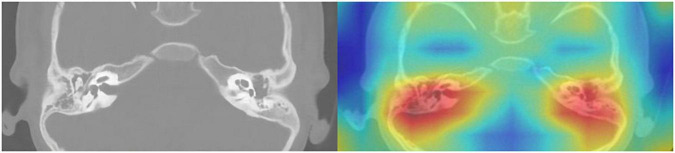
CT of the temporal bone with cholesteatoma: Original image **(left)** and heat map **(right)**.

**FIGURE 4:**
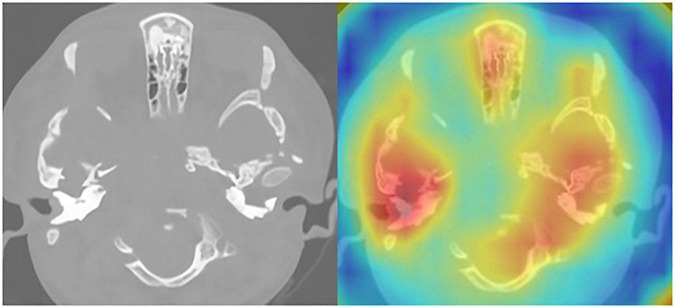
CT of the temporal bone with LCH: Original image **(left)** and heat map **(right)**.

**FIGURE 5:**
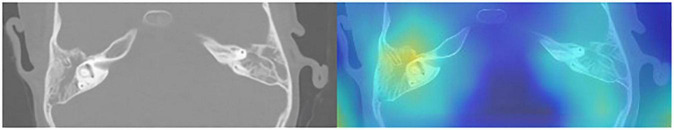
CT of the temporal bone with MEI: Original image **(left)** and heat map **(right)**.

**FIGURE 6:**
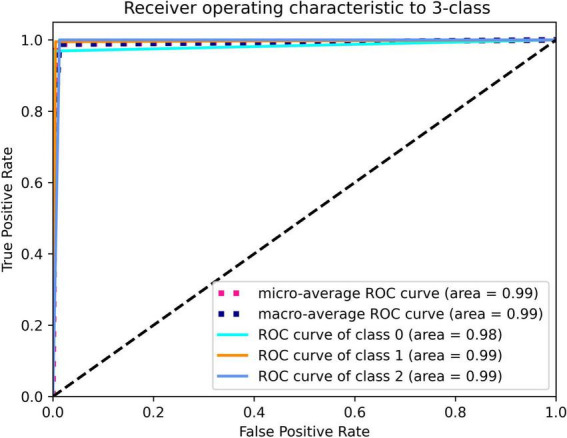
Deep learning framework detection of cholesteatoma (class 0), LCH (class 1), and MEI (class 2).

The model that was adopted in the current study is used in discrete networks. For multivariate classification tasks, the test results were satisfactory when the dataset was tested for the classification of cholesteatoma, LCH, and MEI. Based on the total weight parameters included in the pretraining, the heat map was created according to gradient-weighted class activation mapping. The heat map indicated the differences related to where the computer focused the imaging scan, in which the orange area was significant (Figures 3–5). The area under the curve for this network was 0.99 (see Figure 6). The network derived a better conclusion than the four experts who were included in the clinical trial.

### Classification network and clinical experts’ performance

The training accuracy was 99%, and the validation accuracy was 98.7% at its most balanced time. The confusion matrix is shown in Table 1.

**TABLE 1:** Multi-classification confusion matrix for deep learning.

	Predict cholesteatoma	LCH	MEI
Cholesteatoma	185	2	4
LCH	0	190	1
MEI	0	0	191

The overall accuracy of the network was high (98.7 vs. 88.5%) for the three task categories, and the network achieved a higher F1 score (98.7 vs. 88.2%) compared to the clinical experts. These results are shown in Table 2.

**TABLE 2:** Confusion matrix comparison of AI vs. clinical experts.

	Precision	Recall	F1 score	Accuracy
Clinical experts	0.906	0.885	0.882	0.885
vgg16_bn	0.988	0.987	0.987	0.987

## Discussion

With the rapid development of deep learning technology and the use of big data to develop the training of these networks, healthcare AI technology has undergone rapid development. Radiomics analysis of different types of imaging data has yielded valuable results that can be used to isolate different tumor entities throughout the human body. In some areas that rely on medical imaging, particularly radiology and pathology, the use of machine learning methods to perform a variety of medical tasks (and to a higher quality than physicians) has been very satisfactory. For example, machine learning is currently used in CT scanning to detect abnormalities in the head, classify masses in the liver, and predict amyotrophic lateral sclerosis. In the fields of neurology and otology, many leading figures have applied basic machine learning techniques for predicting the hearing results of patients experiencing sudden hearing impairment (9), or to make predictions regarding the hearing and language perception of children with cochlear implants (10).

The current article is the first study to apply a deep learning network to temporal bone tumors in children, based on CT images. The performance of the adopted model was excellent, and its sensitivity and specificity were superior to those of medical and clinical experts. These advantages, as well as the absolute consistency and high efficiency of AI in decision-making, indicated that the clinical application of AI in the diagnosis of temporal bone tumors, based on CT scans, represents broad development prospects.

In the field of deep learning, overfitting is a classic problem, particularly when the number of samples is limited or the training steps are extensive.

In response to the problem of overfitting, the following methods can be used to avoid problems and improve the versatility of a model. Using data enhancement technology, the application of this technology can greatly increase the number of images that can be used for network training. A network model can use this method to achieve 98, 99, and 99% accuracy in the three classification tasks (training, verifying, and testing datasets, respectively).

The accurate diagnosis of otitis media is a challenging task. The key factor for a correct diagnosis in this regard is using guidelines and practices that are currently implemented by the country in which the research is conducted. This includes accurately confirming the length of time of infection and determining each TM that expands or contracts.

Acquired cholesteatoma has a history dating back three centuries, but the nature of this disease remains unconfirmed. If cholesteatoma is not detected and treated in time, it can become very dangerous by rapidly expanding and invading the internal structure of the temporal bone, causing various intra- and extracranial complications.

As shown in Figure 1, according to a CT scan, a diagnosis of otitis media may be assumed. However, the patient in question actually suffered from LCH, the diagnosis of which is histology-based.

It is difficult to identify a temporal mass using the naked eye without the presence of bone damage. With this model, an early diagnosis can be made before the temporal bone is entirely destroyed.

Following further analysis and inspection of misclassified cases, the network logic can be observed. Clinical experts and AI models may attempt to classify both the typical and non-challenging cases. However, in some situations, neither may be able to correctly classify these cases. When this happens, CT scanning will be required to highlight atypical and extremely subtle patterns. Although the strategy that was applied in the AI classification tasks was not clear, when classifying cases of cholesteatoma, LCH, and MEI, the higher recall rate that was obtained reflected that AI was in the CT image. When reading abnormal patterns, there was a high level of sensitivity. This ability is of great value in terms of providing a first step in the screening and diagnosis process because it minimizes the risk of overlooked cases. However, when clinical experts correctly identified more of the normal ears, they proved their greater particularity. Accordingly, to a large extent, they can help eliminate case results that have been misclassified and analyzed by AI to achieve the goal with minimal labor costs.

One of the most important obstacles to the broad application of deep learning in medicine is the inability to understand the basic principles of algorithmic conclusions. In a recent research experiment (11), the researchers constructed a framework based on algorithmic components that could generate a tissue image in a digital optical coherence tomography scan using a segmented network while concurrently creating a classification network; this network was then used for establishing a diagnosis and for providing treatment recommendations. This workflow divided the decision-making process into two distinct steps, i.e., explaining the initial scan, and establishing a judgment, based on the findings of the first step. In the current study, the authors did not design an automatic separation framework. Tumors may not always be confined to the mastoid process and, as such, may involve other parts of the temporal bone. The clinician should, thus, inspect and extract particular regions of interest.

The present study showed that an automated system, developed using deep learning methods, could be useful for identifying otitis media. Various CT imaging features were quantitatively evaluated by applying image processing methods, including segmentation and image feature extraction. The deep learning classifiers used image features to distinguish patients with temporal bone tumors from those with otitis media. This study was designed to examine the value of an automatic method, based on deep learning, for determining patient candidates for tympanotomy.

Employing deep learning methods may expand the number of candidates for tympanotomy. The findings of this study concurred with recent research on a deep learning approach (12), in which machine-learning models were developed to classify patients with chronic otitis media and cholesteatoma. The classifier and an optimal area that was less than 0.99, sensitivity was 0.97, and specificity was 0.80. The accuracy rate of the AI model was approximately 90%, but the average accuracy rate of clinical experts was relatively low (approximately 73.8%).

In the research process, the AI model obtained very good results. The accuracy of the model classification (99%) in the presence of otitis media and temporal bone tumors was 0.99, which was equivalent to/better than the results reported in studies based on ear mirroring, which ranged from 80 to 85.6% (13, 14).

The deep learning model presented in the current article has several possible advantages. The system used a computerized method to quantitatively evaluate various CT parameters. Multiple image features may enhance the ability of the model to predict pathological tissue. Pretrained deep learning models may significantly reduce the time required to determine whether symptom onset had been within the therapeutic time window. These characteristics may enable an otolaryngologist to determine whether patients require tympanotomy or another type of intervention.

The following limitations are present in the current study and must be noted. First, only moderate datasets were available for inclusion, and these temporal tumor samples were too small to support creating a complete classifier, or there was a slight imbalance. Therefore, based on the currently known properties of deep learning techniques, the AI model presented herein can be used in two-dimensional CT images. Two-dimensional CT images are less detailed than their three-dimensional (3D) conunterparts because the latter includes the temporal bone and the middle ear region. Accordingly, for the validated AI model, using 3D images will improve the model’s diagnostic accuracy. The above algorithm is also consistent with the CT scan explained by the clinician.

All the study participants were sourced from the same clinical center. Further verification resulting from multicenter research settings and different patient populations is warranted. Furthermore, the scientific data employed in the current article had been optimized and, accordingly, showed only the current population. As such, the results of this study should be interpreted with care.

Compared with the conclusions derived by the deep learning model, the outcomes derived by clinicians may vary too much for the former to be considered the gold standard. Additional validation using a large database is, thus, necessary.

## Conclusion

The purpose of this study was to establish a deep learning research framework for the diagnosis of LCH of the temporal bone, cholesteatoma, and MEI, based on CT scanning of the temporal bone in children. An in-depth research framework, based on CT scanning of the temporal bone in children, was proposed for the in-depth study of temporal bone fractures, cholesteatoma, and confirming a temporal lobe tumor diagnosis. The results of the study also showed that the clinical application of AI in CT imaging presented good development prospects. In the future, temporal bone research, as well as 3D CT imaging technology, will be used to expand this method to the larger-scale treatment of temporal bone diseases.

## Data availability statement

The raw data supporting the conclusions of this article will be made available by the authors, without undue reservation.

## Ethics statement

The studies involving human participants were reviewed and approved by the Children’s Hospital of Fudan University. Written informed consent to participate in this study was provided by the participants’ legal guardian/next of kin.

## Author contributions

BD and Z-MX: conception and design of the research. BD, L-LP, W-XC, and Z-WQ: acquisition of data. BD, L-LP, and W-XC: analysis and interpretation of the data. BD, Z-WQ, and Z-MX: statistical analysis. BD: writing of the manuscript. Z-MX: critical revision of the manuscript for intellectual content. All authors read and approved the final draft.
